# *N*′-[(1*E*)-3-Bromo-5-chloro-2-hy­droxy­benzyl­idene]-4-*tert*-butyl­benzo­hydrazide ethanol monosolvate

**DOI:** 10.1107/S160053681104027X

**Published:** 2011-10-05

**Authors:** A. Thirugnanasundar, K. Parthipan, V. S. Xavier Anthonisamy, G. Chakkaravarthi, G. Rajagopal

**Affiliations:** a Department of Chemistry, Velalar College of Engineering and Technology, Erode 638 009, India; b Department of Chemistry, Pondicherry University, Pondicherry 605014, India; c Department of Chemistry, Government Arts College, Melur 625 106, India; d Department of Physics, CPCL Polytechnic College, Chennai 600 068, India

## Abstract

In the title compound, C_18_H_18_BrClN_2_O_2_·C_2_H_6_O, the hy­droxy group forms an intra­molecular O—H⋯N hydrogen bond, which influences the conformation of the Shiff base mol­ecule, where the two aromatic rings form a dihedral angle of 21.67 (8)°. Inter­molecular N—H⋯O and O—H⋯O hydrogen bonds link two Shiff base mol­ecules and two solvent mol­ecules into a centrosymmetric heterotetra­mer. Weak inter­molecular C—H⋯O inter­actions link further tetra­mers related by translation along the *a* axis into chains.

## Related literature

For the biological activity of Schiff base derivatives, see: Dao *et al.* (2000 ▶); Karthikeyan *et al.* (2006 ▶); Prabhakaran *et al.* (2006 ▶); Shivakumar *et al.* (2008 ▶). For related structures, see: Fun *et al.* (2008 ▶); Thirugnanasundar *et al.* (2011 ▶).
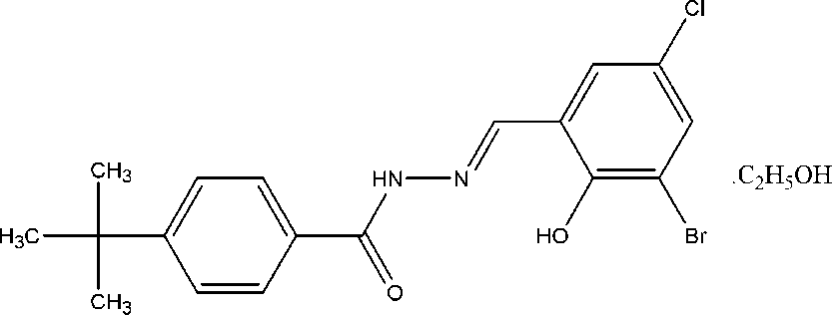


## Experimental

### 

#### Crystal data

C_18_H_18_BrClN_2_O_2_·C_2_H_6_O
*M* *_r_* = 455.77
Triclinic, 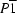

*a* = 9.2478 (4) Å
*b* = 9.4057 (4) Å
*c* = 12.7838 (5) Å
α = 78.811 (2)°
β = 79.235 (1)°
γ = 79.469 (2)°
*V* = 1059.17 (8) Å^3^
*Z* = 2
Mo *K*α radiation
μ = 2.09 mm^−1^
*T* = 295 K
0.26 × 0.22 × 0.20 mm

#### Data collection

Bruker Kappa APEXII diffractometer
Absorption correction: multi-scan (*SADABS*; Sheldrick, 1996 ▶) *T*_min_ = 0.613, *T*_max_ = 0.680
27712 measured reflections
6728 independent reflections
3460 reflections with *I* > 2σ(*I*)
*R*_int_ = 0.033

#### Refinement

*R*[*F* ^2^ > 2σ(*F* ^2^)] = 0.052
*wR*(*F* ^2^) = 0.180
*S* = 1.03
6728 reflections
248 parameters
1 restraint
H-atom parameters constrained
Δρ_max_ = 0.60 e Å^−3^
Δρ_min_ = −0.63 e Å^−3^

### 

Data collection: *APEX2* (Bruker, 2004 ▶); cell refinement: *SAINT* (Bruker, 2004 ▶); data reduction: *SAINT*; program(s) used to solve structure: *SHELXS97* (Sheldrick, 2008 ▶); program(s) used to refine structure: *SHELXL97* (Sheldrick, 2008 ▶); molecular graphics: *PLATON* (Spek, 2009 ▶); software used to prepare material for publication: *SHELXL97*.

## Supplementary Material

Crystal structure: contains datablock(s) global, I. DOI: 10.1107/S160053681104027X/cv5157sup1.cif

Structure factors: contains datablock(s) I. DOI: 10.1107/S160053681104027X/cv5157Isup2.hkl

Supplementary material file. DOI: 10.1107/S160053681104027X/cv5157Isup3.cml

Additional supplementary materials:  crystallographic information; 3D view; checkCIF report

## Figures and Tables

**Table 1:** Hydrogen-bond geometry (Å, °)

*D*—H⋯*A*	*D*—H	H⋯*A*	*D*⋯*A*	*D*—H⋯*A*
O1—H1⋯N1	0.82	1.86	2.577 (3)	145
N2—H2⋯O3	0.86	2.10	2.865 (3)	147
O3—H3⋯O2^i^	0.82	1.94	2.755 (3)	171
C14—H14⋯O2^ii^	0.93	2.54	3.418 (3)	157

Symmetry codes: (i) 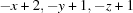; (ii) 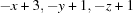.

**Fig. 1.**
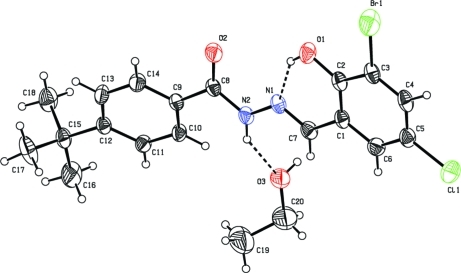
The molecular structure of (I), with atom labels and 30% probability displacement ellipsoids for non-H atoms. Dashed lines denote hydrogen bonds.

**Fig. 2.**
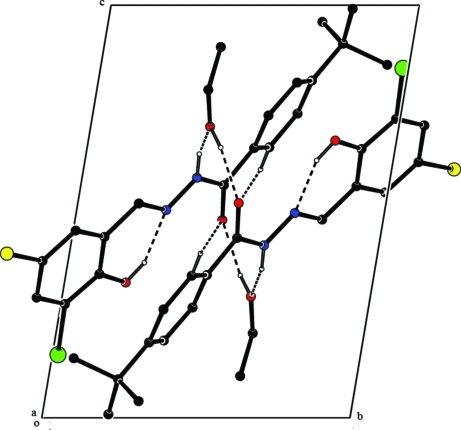
Hydrogen-bonded (dashed lines) tetramer in (I), viewed down a axis. H atoms not involved in hydrogen bonding have been omitted.

## supplementary crystallographic information

### Comment

Schiff base compounds have been widely used as versatile ligands in coordination chemistry (Shivakumar *et al.*, 2008; Prabhakaran *et al.*, 2006). Schiff bases are also known to exhibit anticancer, antibacterial and antifungal activities (Dao *et al.*, 2000; Karthikeyan *et al.*, 2006). Herewith we present the title compound (I), which is a new Schiff base.

In (I) (Fig. 1), the geometric parameters are comparable with the those reported for similar structures ( Fun *et al.*, 2008; Thirugnanasundar *et al.*, 2011). The dihedral angle between the two aromatic rings is  21.67 (8)°. Intermolecular N—H···O and O—H···O hydrogen bonds (Table 1) link two Shiff base molecules and two solvent molecules into centrosymmetric tetramer (Fig. 2). Weak intermolecular C—H···O interactions (Table 1) link further the tetramers related by translation along axis *a* into chains.

### Experimental

4-*tert*-Butylbenzoichydrazide (5 mmol) was dissolved in 20 mL of dry ethanol with stirring and warming over a period of 10 min. To the warm hydrazide solution, 3-bromo-5-chloro salicylaldehyde (5 mmol) in 20 mL of dry ethanol was added and the mixture was stirred and slowly refluxed for 2 h. The mixture was then cooled down to room temperature when pale yellow crystalline compound precipitated. The compound was collected by filtration, washed with cold ethanol and dried in vacuum. Single crystals suitable for the X-ray diffraction were obtained by slow evaporation of a solution of the title compound in ethanol at room temperature. Melting Point: 498 - 500 K.

### Refinement

All H atoms were positioned geometrically [C—H = 0.93-0.97)Å, O—H = 0.82 Å, N—H = 0.86 Å] and allowed to ride on their parent atoms, with U_iso_(H) = 1.2-1.5 U_eq_ of the parent atom. The bond distance C19-C20 was restrained to 1.540 (1)Å.

### Crystal data

**Table:**

C_18_H_18_BrClN_2_O_2_·C_2_H_6_O	*Z* = 2
*M**_r_* = 455.77	*F*(000) = 468
Triclinic, *P*1	*D*_x_ = 1.429 Mg m^−^^3^
Hall symbol: -P 1	Mo *K*α radiation, λ = 0.71073 Å
*a* = 9.2478 (4) Å	Cell parameters from 5983 reflections
*b* = 9.4057 (4) Å	θ = 2.2–25.3°
*c* = 12.7838 (5) Å	µ = 2.09 mm^−^^1^
α = 78.811 (2)°	*T* = 295 K
β = 79.235 (1)°	Block, pale yellow
γ = 79.469 (2)°	0.26 × 0.22 × 0.20 mm
*V* = 1059.17 (8) Å^3^	

### Data collection

**Table:**

Bruker Kappa APEXII diffractometer	6728 independent reflections
Radiation source: fine-focus sealed tube	3460 reflections with *I* > 2σ(*I*)
graphite	*R*_int_ = 0.033
ω and φ scans	θ_max_ = 31.1°, θ_min_ = 2.3°
Absorption correction: multi-scan (*SADABS*; Sheldrick, 1996)	*h* = −13→13
*T*_min_ = 0.613, *T*_max_ = 0.680	*k* = −13→13
27712 measured reflections	*l* = −18→18

### Refinement

**Table:**

Refinement on *F*^2^	Primary atom site location: structure-invariant direct methods
Least-squares matrix: full	Secondary atom site location: difference Fourier map
*R*[*F*^2^ > 2σ(*F*^2^)] = 0.052	Hydrogen site location: inferred from neighbouring sites
*wR*(*F*^2^) = 0.180	H-atom parameters constrained
*S* = 1.03	*w* = 1/[σ^2^(*F*_o_^2^) + (0.0879*P*)^2^ + 0.2447*P*] where *P* = (*F*_o_^2^ + 2*F*_c_^2^)/3
6728 reflections	(Δ/σ)_max_ < 0.001
248 parameters	Δρ_max_ = 0.60 e Å^−^^3^
1 restraint	Δρ_min_ = −0.63 e Å^−^^3^

### Fractional atomic coordinates and isotropic or equivalent isotropic displacement parameters (Å^2^)

**Table:**

	*x*	*y*	*z*	*U*_iso_*/*U*_eq_	
Br1	0.93954 (5)	0.98169 (6)	0.84703 (3)	0.1019 (2)	
Cl1	0.49673 (11)	1.20251 (9)	0.60211 (8)	0.0842 (3)	
O1	1.0463 (2)	0.8013 (2)	0.67203 (17)	0.0626 (5)	
H1	1.0757	0.7517	0.6239	0.094*	
O2	1.2797 (2)	0.5219 (2)	0.52053 (15)	0.0637 (5)	
N1	1.0370 (2)	0.7096 (2)	0.49638 (17)	0.0473 (5)	
N2	1.1026 (2)	0.6271 (2)	0.41810 (17)	0.0465 (5)	
H2	1.0646	0.6326	0.3607	0.056*	
C1	0.8540 (3)	0.8951 (3)	0.5612 (2)	0.0467 (6)	
C2	0.9213 (3)	0.8912 (3)	0.6516 (2)	0.0484 (6)	
C3	0.8529 (3)	0.9868 (3)	0.7243 (2)	0.0549 (7)	
C4	0.7247 (3)	1.0821 (3)	0.7087 (2)	0.0575 (7)	
H4	0.6820	1.1453	0.7575	0.069*	
C5	0.6601 (3)	1.0831 (3)	0.6197 (2)	0.0560 (7)	
C6	0.7235 (3)	0.9919 (3)	0.5463 (2)	0.0545 (6)	
H6	0.6791	0.9948	0.4862	0.065*	
C7	0.9190 (3)	0.8017 (3)	0.4805 (2)	0.0504 (6)	
H7	0.8762	0.8085	0.4191	0.060*	
C8	1.2312 (3)	0.5359 (3)	0.43593 (19)	0.0448 (5)	
C9	1.3105 (3)	0.4581 (2)	0.34618 (19)	0.0413 (5)	
C10	1.2420 (3)	0.4292 (3)	0.2672 (2)	0.0461 (5)	
H10	1.1405	0.4603	0.2676	0.055*	
C11	1.3245 (3)	0.3540 (3)	0.1874 (2)	0.0477 (6)	
H11	1.2765	0.3344	0.1353	0.057*	
C12	1.4758 (3)	0.3071 (2)	0.1826 (2)	0.0444 (5)	
C13	1.5416 (3)	0.3359 (3)	0.2627 (2)	0.0538 (6)	
H13	1.6432	0.3051	0.2620	0.065*	
C14	1.4614 (3)	0.4091 (3)	0.3443 (2)	0.0543 (6)	
H14	1.5090	0.4254	0.3980	0.065*	
C15	1.5690 (3)	0.2267 (3)	0.0933 (2)	0.0539 (6)	
C16	1.4789 (5)	0.2141 (5)	0.0085 (3)	0.0977 (13)	
H16A	1.4379	0.3103	−0.0238	0.146*	
H16B	1.3995	0.1593	0.0418	0.146*	
H16C	1.5423	0.1646	−0.0461	0.146*	
C17	1.7001 (5)	0.3051 (4)	0.0402 (3)	0.0918 (13)	
H17A	1.6648	0.4066	0.0164	0.138*	
H17B	1.7520	0.2610	−0.0208	0.138*	
H17C	1.7664	0.2971	0.0912	0.138*	
C18	1.6298 (4)	0.0714 (3)	0.1443 (3)	0.0780 (10)	
H18A	1.6936	0.0220	0.0898	0.117*	
H18B	1.5484	0.0184	0.1756	0.117*	
H18C	1.6854	0.0765	0.1995	0.117*	
O3	0.8792 (3)	0.6125 (3)	0.29413 (18)	0.0786 (7)	
H3	0.8295	0.5667	0.3451	0.118*	
C19	0.9211 (6)	0.6211 (10)	0.1011 (4)	0.163 (3)	
H19A	0.9967	0.6814	0.0944	0.244*	
H19B	0.8696	0.6507	0.0400	0.244*	
H19C	0.9665	0.5204	0.1040	0.244*	
C20	0.8100 (5)	0.6384 (8)	0.2050 (3)	0.1222 (18)	
H20A	0.7549	0.7370	0.1978	0.147*	
H20B	0.7393	0.5705	0.2149	0.147*	

### Atomic displacement parameters (Å^2^)

**Table:**

	*U*^11^	*U*^22^	*U*^33^	*U*^12^	*U*^13^	*U*^23^
Br1	0.0838 (3)	0.1505 (4)	0.0874 (3)	0.0068 (3)	−0.0185 (2)	−0.0770 (3)
Cl1	0.0797 (6)	0.0691 (5)	0.0899 (6)	0.0249 (4)	−0.0068 (5)	−0.0198 (4)
O1	0.0522 (11)	0.0749 (12)	0.0641 (12)	0.0052 (9)	−0.0073 (9)	−0.0354 (10)
O2	0.0541 (11)	0.0963 (15)	0.0461 (10)	−0.0032 (10)	−0.0084 (9)	−0.0323 (10)
N1	0.0503 (12)	0.0485 (11)	0.0446 (11)	−0.0085 (9)	0.0045 (9)	−0.0216 (9)
N2	0.0493 (12)	0.0500 (11)	0.0411 (11)	−0.0020 (9)	0.0002 (9)	−0.0220 (9)
C1	0.0482 (13)	0.0412 (12)	0.0482 (14)	−0.0070 (10)	0.0069 (11)	−0.0151 (10)
C2	0.0495 (14)	0.0473 (13)	0.0482 (14)	−0.0119 (11)	0.0062 (11)	−0.0169 (11)
C3	0.0543 (15)	0.0617 (15)	0.0511 (15)	−0.0137 (12)	0.0077 (12)	−0.0259 (12)
C4	0.0598 (17)	0.0504 (14)	0.0588 (16)	−0.0089 (12)	0.0159 (13)	−0.0242 (12)
C5	0.0571 (16)	0.0425 (13)	0.0598 (17)	0.0001 (11)	0.0065 (13)	−0.0101 (11)
C6	0.0605 (17)	0.0486 (13)	0.0510 (15)	−0.0019 (12)	−0.0039 (12)	−0.0104 (11)
C7	0.0589 (16)	0.0480 (13)	0.0443 (13)	−0.0057 (11)	−0.0001 (11)	−0.0176 (11)
C8	0.0428 (13)	0.0531 (13)	0.0409 (13)	−0.0103 (10)	0.0002 (10)	−0.0170 (10)
C9	0.0417 (12)	0.0429 (11)	0.0392 (12)	−0.0056 (9)	−0.0007 (9)	−0.0129 (9)
C10	0.0396 (12)	0.0534 (13)	0.0459 (13)	0.0008 (10)	−0.0040 (10)	−0.0201 (11)
C11	0.0468 (14)	0.0540 (13)	0.0447 (13)	0.0002 (10)	−0.0079 (11)	−0.0208 (11)
C12	0.0454 (13)	0.0399 (11)	0.0456 (13)	−0.0036 (9)	0.0018 (10)	−0.0129 (10)
C13	0.0358 (12)	0.0667 (16)	0.0591 (16)	0.0023 (11)	−0.0039 (11)	−0.0235 (13)
C14	0.0455 (14)	0.0695 (16)	0.0521 (15)	−0.0036 (12)	−0.0103 (11)	−0.0226 (13)
C15	0.0535 (15)	0.0503 (13)	0.0554 (15)	−0.0023 (11)	0.0069 (12)	−0.0226 (12)
C16	0.093 (3)	0.124 (3)	0.082 (2)	0.017 (2)	−0.009 (2)	−0.068 (2)
C17	0.099 (3)	0.075 (2)	0.091 (3)	−0.0283 (19)	0.048 (2)	−0.0345 (19)
C18	0.077 (2)	0.0517 (16)	0.095 (3)	0.0024 (14)	0.0109 (19)	−0.0212 (16)
O3	0.0644 (14)	0.1174 (19)	0.0548 (12)	−0.0080 (13)	−0.0132 (10)	−0.0176 (12)
C19	0.104 (4)	0.311 (10)	0.079 (3)	−0.029 (5)	−0.018 (3)	−0.046 (5)
C20	0.096 (3)	0.195 (6)	0.079 (3)	−0.014 (3)	−0.029 (3)	−0.027 (3)

### Geometric parameters (Å, °)

**Table:**

Br1—C3	1.880 (3)	C12—C13	1.376 (4)
Cl1—C5	1.733 (3)	C12—C15	1.532 (3)
O1—C2	1.338 (3)	C13—C14	1.385 (4)
O1—H1	0.8200	C13—H13	0.9300
O2—C8	1.221 (3)	C14—H14	0.9300
N1—C7	1.285 (3)	C15—C17	1.516 (4)
N1—N2	1.369 (3)	C15—C16	1.519 (5)
N2—C8	1.362 (3)	C15—C18	1.530 (4)
N2—H2	0.8601	C16—H16A	0.9600
C1—C6	1.392 (4)	C16—H16B	0.9600
C1—C2	1.404 (4)	C16—H16C	0.9600
C1—C7	1.457 (3)	C17—H17A	0.9600
C2—C3	1.402 (3)	C17—H17B	0.9600
C3—C4	1.371 (4)	C17—H17C	0.9600
C4—C5	1.378 (4)	C18—H18A	0.9600
C4—H4	0.9300	C18—H18B	0.9600
C5—C6	1.372 (4)	C18—H18C	0.9600
C6—H6	0.9300	O3—C20	1.369 (5)
C7—H7	0.9300	O3—H3	0.8201
C8—C9	1.485 (3)	C19—C20	1.5362 (10)
C9—C10	1.379 (3)	C19—H19A	0.9600
C9—C14	1.385 (4)	C19—H19B	0.9600
C10—C11	1.384 (3)	C19—H19C	0.9600
C10—H10	0.9300	C20—H20A	0.9700
C11—C12	1.381 (4)	C20—H20B	0.9700
C11—H11	0.9300		
			
C2—O1—H1	109.5	C12—C13—H13	118.9
C7—N1—N2	118.4 (2)	C14—C13—H13	118.9
C8—N2—N1	116.5 (2)	C13—C14—C9	120.0 (2)
C8—N2—H2	121.8	C13—C14—H14	120.0
N1—N2—H2	121.8	C9—C14—H14	120.0
C6—C1—C2	119.7 (2)	C17—C15—C16	109.8 (3)
C6—C1—C7	118.7 (3)	C17—C15—C18	108.2 (3)
C2—C1—C7	121.5 (2)	C16—C15—C18	107.9 (3)
O1—C2—C3	118.6 (3)	C17—C15—C12	109.6 (2)
O1—C2—C1	123.5 (2)	C16—C15—C12	112.6 (2)
C3—C2—C1	117.9 (2)	C18—C15—C12	108.6 (2)
C4—C3—C2	121.9 (3)	C15—C16—H16A	109.5
C4—C3—Br1	119.4 (2)	C15—C16—H16B	109.5
C2—C3—Br1	118.6 (2)	H16A—C16—H16B	109.5
C3—C4—C5	119.1 (2)	C15—C16—H16C	109.5
C3—C4—H4	120.4	H16A—C16—H16C	109.5
C5—C4—H4	120.4	H16B—C16—H16C	109.5
C6—C5—C4	120.9 (3)	C15—C17—H17A	109.5
C6—C5—Cl1	120.5 (3)	C15—C17—H17B	109.5
C4—C5—Cl1	118.6 (2)	H17A—C17—H17B	109.5
C5—C6—C1	120.4 (3)	C15—C17—H17C	109.5
C5—C6—H6	119.8	H17A—C17—H17C	109.5
C1—C6—H6	119.8	H17B—C17—H17C	109.5
N1—C7—C1	118.3 (2)	C15—C18—H18A	109.5
N1—C7—H7	120.8	C15—C18—H18B	109.5
C1—C7—H7	120.8	H18A—C18—H18B	109.5
O2—C8—N2	121.4 (2)	C15—C18—H18C	109.5
O2—C8—C9	122.2 (2)	H18A—C18—H18C	109.5
N2—C8—C9	116.4 (2)	H18B—C18—H18C	109.5
C10—C9—C14	118.8 (2)	C20—O3—H3	109.6
C10—C9—C8	123.9 (2)	C20—C19—H19A	109.5
C14—C9—C8	117.3 (2)	C20—C19—H19B	109.5
C9—C10—C11	119.9 (2)	H19A—C19—H19B	109.5
C9—C10—H10	120.0	C20—C19—H19C	109.5
C11—C10—H10	120.0	H19A—C19—H19C	109.5
C12—C11—C10	122.3 (2)	H19B—C19—H19C	109.5
C12—C11—H11	118.9	O3—C20—C19	112.2 (4)
C10—C11—H11	118.9	O3—C20—H20A	109.2
C13—C12—C11	116.8 (2)	C19—C20—H20A	109.2
C13—C12—C15	120.4 (2)	O3—C20—H20B	109.2
C11—C12—C15	122.8 (2)	C19—C20—H20B	109.2
C12—C13—C14	122.1 (2)	H20A—C20—H20B	107.9
			
C7—N1—N2—C8	177.0 (2)	N1—N2—C8—C9	−173.9 (2)
C6—C1—C2—O1	179.8 (2)	O2—C8—C9—C10	157.1 (3)
C7—C1—C2—O1	−1.4 (4)	N2—C8—C9—C10	−24.4 (4)
C6—C1—C2—C3	0.0 (4)	O2—C8—C9—C14	−21.3 (4)
C7—C1—C2—C3	178.8 (2)	N2—C8—C9—C14	157.2 (2)
O1—C2—C3—C4	179.9 (2)	C14—C9—C10—C11	−0.7 (4)
C1—C2—C3—C4	−0.3 (4)	C8—C9—C10—C11	−179.0 (2)
O1—C2—C3—Br1	−0.6 (3)	C9—C10—C11—C12	−0.8 (4)
C1—C2—C3—Br1	179.15 (19)	C10—C11—C12—C13	1.3 (4)
C2—C3—C4—C5	0.8 (4)	C10—C11—C12—C15	−178.6 (2)
Br1—C3—C4—C5	−178.6 (2)	C11—C12—C13—C14	−0.4 (4)
C3—C4—C5—C6	−1.0 (4)	C15—C12—C13—C14	179.5 (3)
C3—C4—C5—Cl1	179.1 (2)	C12—C13—C14—C9	−1.1 (4)
C4—C5—C6—C1	0.8 (4)	C10—C9—C14—C13	1.6 (4)
Cl1—C5—C6—C1	−179.4 (2)	C8—C9—C14—C13	−180.0 (3)
C2—C1—C6—C5	−0.3 (4)	C13—C12—C15—C17	−53.7 (4)
C7—C1—C6—C5	−179.1 (2)	C11—C12—C15—C17	126.2 (3)
N2—N1—C7—C1	−176.7 (2)	C13—C12—C15—C16	−176.2 (3)
C6—C1—C7—N1	−178.0 (2)	C11—C12—C15—C16	3.7 (4)
C2—C1—C7—N1	3.2 (4)	C13—C12—C15—C18	64.3 (3)
N1—N2—C8—O2	4.6 (4)	C11—C12—C15—C18	−115.8 (3)

### Hydrogen-bond geometry (Å, °)

**Table:**

*D*—H···*A*	*D*—H	H···*A*	*D*···*A*	*D*—H···*A*
O1—H1···N1	0.82	1.86	2.577 (3)	145
N2—H2···O3	0.86	2.10	2.865 (3)	147
O3—H3···O2^i^	0.82	1.94	2.755 (3)	171
C14—H14···O2^ii^	0.93	2.54	3.418 (3)	157

Symmetry codes: (i) −*x*+2, −*y*+1, −*z*+1; (ii) −*x*+3, −*y*+1, −*z*+1.
